# Association of Plasminogen Activator Inhibitor-1 (*PAI-1*) Gene Polymorphisms with Osteoporotic Vertebral Compression Fractures (OVCFs) in Postmenopausal Women

**DOI:** 10.3390/ijms17122062

**Published:** 2016-12-09

**Authors:** Jung Oh Kim, Soo Hong Han, Yeon Ho Lee, Tae Keun Ahn, Jae Joon Lim, Young Sun Chung, Dong Eun Shin, Woo Sik Lee, In Bo Han, Nam Keun Kim

**Affiliations:** 1 Department of Biomedical Science, College of Life Science, CHA University, Seongnam 13488, Korea; jokim8505@gamil.com; 2 Department of Orthopedics, CHA Bundang Medical Center, CHA University, Seongnam 13496, Korea; hsoohong@cha.ac.kr (S.H.H.); onho1@medigate.net (Y.H.L.); zenos@chamc.co.kr (T.K.A.); shinde@chamc.co.kr (D.E.S.); 3 Department of Neurosurgery, CHA Bundang Medical Center, CHA University, Seongnam 13496, Korea; coolppeng@naver.com; 4 Department of Neurosurgery, Konkuk University Chungju Hospital, Chungju 27376, Korea; cysns@kku.ac.kr; 5 Fertility Center of CHA Gangnam Medical Center, CHA University, Seoul 06135, Korea; wooslee@cha.ac.kr

**Keywords:** polymorphism, osteoporotic vertebral compression fractures, *PAI-1*, plasminogen activator inhibitor-1, osteoporosis

## Abstract

Osteoporosis and osteoporotic fractures are strongly associated with mortality and morbidity, both in developing and developed countries. Menopause accelerates bone loss due to estrogen deficiency and age-related linear bone loss. We investigated plasminogen activator inhibitor-1 (*PAI-1*) gene polymorphisms in postmenopausal women with osteoporotic vertebral compression fractures (OVCFs). In this case-control study, 355 postmenopausal women were genotyped for the presence of *PAI-1* gene polymorphisms −844A > G, −675 4G > 5G, 43G > A, 9785A > G, and 11053T > G. Genetic polymorphisms of *PAI-1* were analyzed by the polymerization chain reaction restriction fragment length polymorphism assay, and their association with disease status and folate and homocysteine levels was determined in 158 OVCF patients and 197 control subjects. The *PAI-1* −675 5G5G (adjusted odds ratio (AOR), 3.302; *p =* 0.017) and 43GA + AA (AOR, 2.087; *p =* 0.042) genotype frequencies showed significant association with the increased prevalence of OVCFs in postmenopausal women. In addition, we performed gene–environment interaction studies and demonstrated an association between *PAI-1* gene polymorphisms and OVCF prevalence. Our novel finding is the identification of several *PAI-1* genetic variants that increase susceptibility to OVCF. Our findings suggest that polymorphisms in *PAI-1* may contribute to OVCF, and that they can be developed as biomarkers for evaluating OVCF risk.

## 1. Introduction

Osteoporosis is a common metabolic bone disorder characterized by reduced bone mass, increased skeletal fragility, microarchitectural deterioration, and an increase in bone fractures [1]. Osteoporosis leads to decreased skeletal strength and increased fracture susceptibility. Osteoporotic fractures are a leading cause of disability and, subsequently, death in postmenopausal women [1,2]. The occurrence of osteoporotic fractures is closely associated with mortality worldwide [3,4]. Approximately 30% of women and 12% of men will be affected by osteoporosis in their lifetime; therefore, osteoporosis imposes a major economic burden on society. In particular, osteoporosis and osteoporotic fractures occur frequently in postmenopausal women due to decreased estrogen levels [5,6]. Estrogen deficiency associated with menopause, combined with age-related linear bone loss, leads to accelerated bone loss [7]. Bone mineral density (BMD) is a quantitative trait (g/cm^2^), with a normal distribution based on age and sex [8], and is thought to be controlled genetically in 50%–90% of cases [9,10,11,12]. A previous study has shown that several gene polymorphisms can affect BMD levels [6,12,13,14,15,16].

The plasminogen activator inhibitor-1 (*PAI-1*) gene has several alternative names; the Human Gene Nomenclature Committee (HGNC) provides SERPINE1 as the name that is used officially. The *PAI-1* gene encodes a member of the serine protease inhibitor superfamily (located on chr.7q21.3). The PAI-1 protein inhibits plasminogen activators , including tissue plasminogen activator (tPA) and urokinase-type plasminogen activator (uPA), that touch off a fibrinolysis pathway by conversion from plasminogen to plasmin [17]. Following the formation of a fibrin clot, the fibrinolytic system is activated through regulation by *PAI-1*. The fibrinolytic system provides vessel elasticity by eliminating thrombosis, dismantling the extracellular matrix, and causing tissue remodeling, cell adhesion, and cell migration [18,19]. Daci et al. [20] previously reported that PAI-1 deficiency partially protects against bone loss in estrogen-deficient mice. Another study reported prevention of bone loss in diabetes in PAI-1-deficient mice [21,22]. A recent study has shown that plasminogen is involved in bone recovery in mice [22], suggesting that fibrinolysis in tissue might be a crucial factor in bone fracture recovery [23].

Many previous studies have investigated functional PAI-1 in osteoporosis patients [20,21,23]. However, none have reported on the association between *PAI-1* polymorphisms and osteoporotic vertebral compression fractures (OVCFs) in postmenopausal women. Therefore, the purpose of this study was to investigate whether these polymorphisms of *PAI-1* (−844A > G, −675 4G > 5G, 43G > A, 9785A > G, and 11053T > G) correlate with OVCF susceptibility in postmenopausal women.

## 2. Results

Demographic characteristics and clinical profiles of osteoporosis patients (with/without osteoporotic vertebral compression fracture, OVCF) and controls are summarized in Table 1 and Table S1.

### 2.1. Genotype Frequencies of the PAI-1 Polymorphisms

Genotype and allele frequencies of the *PAI-1* polymorphisms −844A > G, −675 4G > 5G, 43G > A, 9785A > G, and 11053T > G are listed for osteoporosis patients (with/without OVCF) and control in Table 2. Table 2 shows association between osteoporosis and *PAI-1* polymorphisms including −675 4G > 5G (4G4G vs. 5G5G: adjusted odds ratio (AOR) 3.302, 95% confidence interval (CI) 1.224–7.512, *p =* 0.017; 4G4G vs. 4G5G + 5G5G: AOR 1.727, 95% CI 1.102–2.706, *p =* 0.017; and 4G4G + 4G5G vs. 5G5G: AOR 2.510, 95% CI 1.056–5.968, *p =* 0.037), and +43G > A (GG vs. GA + AA: AOR 2.087, 95% CI 1.027–4.241, *p =* 0.042). In addition, it shows a significant correlation between OVCF risk and *PAI-1* polymorphisms, −844G > A (GG vs. GA: AOR 2.244, 95% CI 1.164–4.326, *p =* 0.015; GG vs. GA + AA: AOR 1.918, 95% CI 1.016–3.621, *p =* 0.044), −675 4G > 5G (4G4G vs. 5G5G: AOR 4.646, 95% CI 1.625–13.286, *p =* 0.004; 4G4G vs. 4G5G + 5G5G: AOR 1.969, 95% CI 1.130–3.430, *p =* 0.017; 4G4G + 4G5G vs. 5G5G: AOR 3.378, 95% CI 1.301–8.769, *p =* 0.012), and +43 G > A (GG vs. GA: AOR 2.421, 95% CI 1.057–5.546, *p =* 0.017; GG vs. GA + AA: AOR 2.292, 95% CI 1.009–5.206, *p =* 0.048). Furthermore, we analyzed the association between OVCF patients and non-OVCF patients (Table S2).

### 2.2. Haplotype Analysis

The linkage disequilibrium of the *PAI-1* polymorphisms at loci −844/−675/43/9785/11053 in patients with OVCF and those in the control group is shown in Figure S1. There was strong linkage disequilibrium between loci −844 and −675 (D’ = 0.838, LOD = 8.42, *r*^2^ = 0.153), −844 and +43 (D’ = 0.764, LOD = 1.2, *r*^2^ = 0.02) in the control group, whereas OVCF patients showed strong linkage disequilibrium between loci +43 and +11053 (D’ = 1.000, LOD = 4.51, *r*^2^ = 0.106), +43 and +9785 (D’ = 0.831, LOD = 0.06, *r*^2^ = 0.002). Haplotype analysis of five, four, three, and two loci is presented in Table 3 and Tables S3–S5. Five-polymorphism allelic combination analysis resulted in meaningful combination models: G-4G-G-G-G (odds ratio (OR), 2.231; 95% CI, 1.189–4.187; *p =* 0.015), G-4G-A-G-T (OR, 62.33; 95% CI, 3.547–1095; *p <* 0.0001), G-4G-A-G-G (OR, 8.000; 95% CI, 1.996–32.06; *p =* 0.002), G-5G-G-G-T (OR, 2.565; 95% CI, 1.483–4.434; *p =* 0.001), G-5G-G-G-G (OR, 5.571; 95% CI, 2.039–15.22; *p =* 0.001), G-5G-A-G-T (OR, 5.455; 95% CI, 2.353–12.64; *p <* 0.0001), A-4G-G-G-G (OR, 2.195; 95% CI, 1.346–3.580; *p =* 0.002), A-4G-G-A-G (OR, 32.65; 95% CI, 1.754–607.7; *p =* 0.001), A-4G-A-G-G (OR, 45.00; 95% CI, 5.706–354.9; *p* < 0.0001), A-5G-G-G-T (OR, 38.590; 95% CI, 2.112–705.1; *p =* 0.0004), and A-5G-A-G-T (OR, 56.40; 95% CI, 3.188–997.7; *p* < 0.0001) (Table 3). In Table 3, we found difference between non-OVCF and OVCF risk in haplotype analysis. Haplotype analyses of four-, three-, and two-polymorphism allelic combinations demonstrated many statistically significant results, which are listed in Tables S3–S5.

### 2.3. Combined Effects between PAI-1 Polymorphisms and Environmental Factors

**Figure 1:**
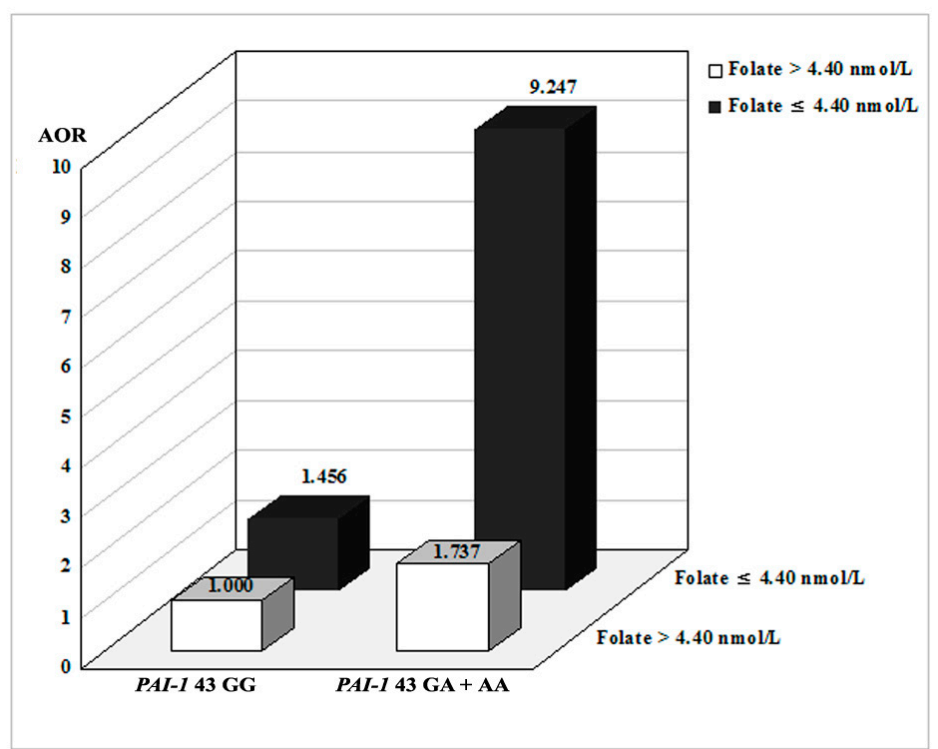
Osteoporosis risk stratified by interaction between *PAI-1* +43G > A and plasma folate levels. Combinations of the *PAI-1* +43 genotype and plasma folate level subgroups. We divided subjects into two groups representing the lower 15% cutoff values for plasma folate levels: >4.40 ng/mL and ≤4.40 ng/mL.

To determine additional clinical significance, we performed stratified (Table S6) and gene–environment interaction (Table S7) analyses according to hypertension, diabetes mellitus, 25-hydroxyl vitamin D (25-OH vit. D), vitamin B_12_, folate, and plasma total homocysteine (tHcy). We divided subjects into two groups representing the upper and lower 15% cutoff values for plasma tHcy and folate levels (folate: 4.40 ng/mL, tHcy: 12.41 µmol/L). We performed stratified analyses comparing clinical factors and polymorphisms (Table S6). Clinical risk factors strongly correlated with *PAI-1* −844 G > A, and −675 4G > 5G polymorphisms. In gene–environment interaction studies (Table S7), the *PAI-1* −675 polymorphism, coupled with low plasma folate levels or high homocysteine levels, was associated with an increased odds ratio when comparing osteoporosis patients with control subjects. In addition, when comparing the association of *PAI-1* +43 and plasma folate levels with osteoporosis (Figure 1), the *PAI-1* +43 GA + AA genotype combined with low plasma folate levels produced a significant result (GG vs. GA + AA: AOR, 9.247; 95% CI, 1.049–81.51). In addition, we evaluated the effects of *PAI-1* genotypes on bone mineral density (BMD), plasma vitamin B_12_, 25-OH vit. D concentrations, and bone metabolism markers (osteocalcin and deoxypyridinoline) in Table S8. *PAI-1* −844G > A was significantly associated with BMD. Reduced osteocalcin and deoxypyridinoline were influenced by *PAI-1* −844GA + AA and 11053TG + GG genotypes, respectively (Figure S2).

## 3. Discussion

Osteoporosis and low BMD commonly occur in postmenopausal women and lead to increased susceptibility to fractures. BMD, along with other factors, is known to contribute to skeletal health [24]. In particular, the role of genetic factors, including gene polymorphisms, takes center stage in pathogenic studies [8,25,26]. Recent studies investigated synergic effects between gene polymorphisms and OVCF risk factors [27,28,29].

PAI-1 is a downregulator of the fibrinolysis gene and the key factor for plasminogen ratios [23,30]. Rundle et al. [31] showed that PAI-1 deficiency results in an increase in not only callus size but also callus cartilage regeneration in PAI-1 knockout (KO) mice recovering from fractures. In addition, fracture callus development was promoted in PAI-1 KO mice in comparison to control mice. These results showed strengthened tissue fibrinolysis in PAI-1 deficiency, which may enhance fracture recovery by facilitating extracellular matrix regeneration [23,31]. This is consistent with our current finding that an abnormal plasminogen level adversely affects bone recovery and regeneration. Furthermore, fibrinolysis in tissue can be a crucial factor in both acute inflammation and fracture restoration [23,32].

A previous study [33] described the 10 most well-known polymorphisms of *PAI-1*, as follows: rs2227631 G > A, rs6092 G > A, rs2227708 C > T, rs2227662 C > T, rs2227666 G > A, rs2227667 A > G, rs2227672 G > T, rs2227683 A > G, rs2227694 G > A, and rs7242 T > G. The rs1799889 (*PAI-1* −675 4G > 5G) polymorphism is widely genotyped in *PAI-1* genetic studies. The five polymorphisms selected for our study affect transcription and are expected to regulate PAI-1 levels. Therefore, we hypothesized that regulation of PAI-1 activity by *PAI-1* polymorphisms results in an abnormality in the bone repair system or bone loss mechanism. Furthermore, postmenopausal women have an increased risk for OVCF because of sex hormone dysregulation. We have identified an association between *PAI-1* polymorphisms and OVCF prevalence. Based on statistical analyses, this result demonstrates a significant correlation between *PAI-1* −844A > G, −675 4G > 5G, and 43G > A, and OVCF risk. However, another study reported the lack of any association between the rs1799889 (*PAI-1* −675 4G > 5G) polymorphism and osteoporosis in Turkish postmenopausal women [34]. Interestingly, this finding conflicts with the results of our research; the discrepancy could be due to differences in the ethnic group or sample size used. On the other hand, the Turkish postmenopausal women exhibited only osteoporosis [35], whereas our study participants exhibited symptoms of greater severity, such as compound fracture due to osteoporosis; this could also be the reason for the discrepancy between the results of the two studies. There are several limitations to our study. First, serum PAI-1 concentrations were not examined in our study population. Previous studies have evaluated the contribution of each *PAI-1* polymorphism to serum PAI-1 expression [35,36,37]. Second, functional studies for *PAI-1* SNP were not performed to elucidate OVCF-related pathogenesis. Although several studies have reported an association between *PAI-1* polymorphisms and bone repair, few have evaluated the pathogenesis by which *PAI-1* polymorphisms affect osteoporosis in postmenopausal women. This study cannot, therefore, propose a detailed pathogenesis by which *PAI-1* polymorphism affects vertebral artery and tissue. Finally, PAI-1 expression depends on the *PAI-1* single nucleotide polymorphism (SNP) genotypes in the local tissue; therefore, PAI-1 expression in vertebral tissue is more important than that in serum.

## 4. Materials and Methods

### 4.1. Study Population

This was a case-control study, in which the case group consisted of 158 osteoporotic postmenopausal women (mean age ± SD, 69.50 ± 8.24 years; age range, 56–81 years), defined as having a *T*-score of −2.5 or lower and/or at least one non-traumatic fracture of the spine. Women were recruited from the Neurosurgery and Orthopedic Surgery Departments at CHA Bundang Medical Center, CHA University of South Korea between March 2005 and December 2008. The control group consisted of 197 postmenopausal women (mean age ± SD, 66.57 ± 8.05 years; age range, 50–85 years) without disease and not taking any medications known to influence bone mass or bone turnover. BMD at the lumbar spine was measured using a dual-energy X-ray (Hologic Discovery W, Waltham, MA, USA). An OVCF was defined as a ≥15% reduction in the anterior, posterior, or central height of the vertebra [38]. All examinations were performed by a trained neurosurgeon and orthopedic surgeon according to previously described methods [39]. All subjects provided their informed consent for inclusion before they participated in the study. The study was conducted in accordance with the Declaration of Helsinki, and the protocol was approved by the Ethics Committee of the Institutional Review Board of CHA Bundang Medical Center on 20 January 2014 (reference No. BD2015-043). We provided each participant with a full explanation of participation in this study and obtained confirmed written consent from all study participants. Bone mineral density (BMD) of the lumbar spine (L2–L4), femur neck (FN), Ward’s triangle, and trochanter (Tro) were measured using dual-energy X-ray absorptiometry (Norland Medical Systems, White Plains, NY, USA). Osteoporosis was defined according to the 1994 classification of the World Health Organization (WHO) [40].

### 4.2. Genotyping

We performed DNA extraction from peripheral blood with an anticoagulant tube using the G-DEX™ II kit (Intron Biotechnology, Seongnam, Korea). The PAI-1 polymorphisms were amplified by polymerase chain reaction and digested with a restriction enzyme to identify them via restriction fragment length polymorphism (RFLP; Table S9) [41]. We selected 30% of the PCR product at random and checked the RFLP results for concordance with DNA sequencing results. The concordance of the quality control samples was 100%.

### 4.3. Bone Measurements

The diagnosis of osteoporosis was based on WHO criteria (dual energy X-ray absorptiometry (DXA) hip or lumbar *T*-score <−2.5 standard deviations), and an OVCF was diagnosed when a progressive or newly generated compression fracture was identified after low-energy trauma. The BMD *T*-score was measured in g/cm^2^ using Hologic densitometers (Hologic Inc., Waltham, MA, USA) [39]. The control group had a BMD *T*-score greater than −1.0 and had no spine or hip fractures based on the results of simple radiography. The BMD *T*-score analysis in this study was conducted with reference to the WHO report [40].

### 4.4. Measurement of Vitamin B_12_, Plasma Total Homocysteine (tHcy), and Folate Levels

We collected plasma samples to measure vitamin B_12_, tHcy, and folate levels within 48 h of osteoporosis detection. Twelve hours after the patient’s previous meal, we collected whole blood in a tube containing anticoagulant. We centrifuged the tube for 15 min at 1000× *g* to separate the plasma and stored it at −80 °C. Plasma vitamin B_12_ concentrations were measured using the BioRad Quantaphase II radioassay (Hercules, CA, USA). We measured plasma tHcy concentration using a fluorescent polarizing immunoassay with the IMx system (Abbott Laboratories, Chicago, IL, USA) and the plasma folate concentration with a radio assay kit (ACS 180; Bayer, Tarrytown, NY, USA).

### 4.5. Statistical Analysis

The genotype and allele combination frequency differences between case and control subjects were analyses by logistic regression and Fisher’s exact test. The adjusted odds ratio (AORs) and confidence intervals (CIs) were used as a measure of the association between genotype frequencies and osteoporosis. All of the alleles were in Hardy–Weinberg equilibrium (*p* > 0.05). The genotypes with osteoporosis and OVCF occurrence was calculated with AORs and 95% CIs from logistic regression adjusted for age, hypertension, and diabetes mellitus. The linkage disequilibrium (LD) with blocking calculation with HaploView software and haplotype analysis for the models chosen by multifactor dimensionality reduction (MDR) methods were checked with the HAPSTAT program version 3.0 (www.bios.unc.edu/~lin/hapstat). The correlations of genotypes with BMD, as well as plasma folate, vitamin B_12_, 25-hydroxyl vitamin D, osteocalcin and deoxypyridinoline levels, were evaluated by Kruskal–Wallis test and Mann–Whitney test. Statistical analyses were performed by GraphPad Prism 4.0 (GraphPad Software Inc., San Diego, CA, USA), MedCalc software version 16.8.4 (MedCalc Ltd., Ostend, Belgium), HaploView 4.1 (Broad Inc., Cambridge, MA, USA), and HAPSTAT 3.0 (Univ. of North Carolina, Chapel Hill, NC, USA).

## 5. Conclusions

In summary, we found an association between *PAI-1* polymorphisms and osteoporosis risk in postmenopausal women. Furthermore, *PAI-1* polymorphisms were shown to strongly association with OVCF occurrence. Interestingly, our results suggest differences between non-OVCF and OVCF patients in this association with *PAI-1* polymorphisms. In particular, the *PAI-1* −844G > A, −675 4G > 5G, +43G > A polymorphisms were associated with increased susceptibility for OVCF rather than non-OVCF and osteoporosis. Therefore, we expect a potentially higher fracture risk in postmenopausal women who are osteoporosis patients with these *PAI-1* polymorphisms. Our findings present the first report on the association between *PAI-1* polymorphisms and OVCF. In conclusion, this study suggests that *PAI-1* polymorphisms (−844G > A, −675 4G > 5G, +43G > A) may contribute to OVCF and could be used as genetic biomarkers for OVCF risk.

## Figures and Tables

**Table 1:** Baseline characteristics between controls and osteoporosis patients.

Characteristic	Controls (*n* = 197)	Osteoporosis (*n* = 158)	*p*	OVCF (*n* = 87)	*p*	Non-OVCF (*n* = 71)	*p*
Age (years, mean ± SD)	66.11 ± 8.93	70.50 ± 8.24	<0.0001	70.23 ± 9.44	0.001	70.83 ± 6.53	<0.0001
Hypertension (%)	92 (46.7)	57 (36.1)	0.196	37 (42.5)	0.760	20 (28.2)	0.087
SBP (mmHg, mean ± SD)	134.98 ± 18.13	128.42 ± 15.27	0.0005	129.99 ± 16.51	0.041	126.66 ± 13.65	0.001
DBP (mmHg, mean ± SD)	80.69 ± 12.22	76.13 ± 10.44	0.0003	77.63 ± 10.57	0.049	74.45 ± 10.10	0.0001
Diabetes mellitus (%)	25 (12.7)	28 (17.7)	0.324	11 (12.6)	0.992	17 (23.9)	0.062
FBS (mg/dL, mean ± SD)	110.46 ± 33.98	125.80 ± 44.48	<0.0001	135.89 ± 53.96	<0.0001	114.30 ± 26.15	0.025
Hcy (μmol/L, mean ± SD)	9.38 ± 2.89	9.81 ± 4.06	0.056	10.01 ± 3.85	0.022	9.57 ± 4.31	0.316
Folate (ng/mL, mean ± SD)	9.67 ± 9.25	8.12 ± 4.78	0.054	6.54 ± 4.03	0.003	10.19 ± 4.91	0.027
BMI (kg/m^2^, mean ± SD)	24.51 ± 3.30	23.88 ± 4.04	0.169	23.87 ± 5.02	0.286	23.89 ± 2.75	0.180
HDL-cholesterol (mg/dL, mean ± SD)	47.83 ± 12.05	44.60 ± 14.24	0.201	44.39 ± 15.95	0.249	44.83 ± 12.24	0.250
LDL-cholesterol (mg/dL, mean ± SD)	131.09 ± 44.47	106.74 ± 39.49	0.001	117.87 ± 42.82	0.161	95.02 ± 32.02	<0.0001
TG (mg/dL, mean ± SD)	150.29 ± 88.98	140.66 ± 80.11	0.324	147.62 ± 74.17	0.824	132.23 ± 86.68	0.176
Vitamin B_12_ (pg/mL, mean ± SD)	788.25 ± 821.74	710.69 ± 591.37	0.015	973.11 ± 1024.37	0.361	639.46 ± 384.93	0.006
25-(OH) Vitamin D (ng/mL, mean ± SD)	-	24.02 ± 21.50	-	41.97 ± 34.20	-	18.57 ± 11.62	-
Osteocalcin (ng/mL, mean ± SD)	-	7.83 ± 6.39	-	7.83 ± 6.39	-	-	-
DPD (nMDP/mMcre, mean ± SD)	-	8.41 ± 5.55	-	8.41 ± 5.55	-	-	-
BMD (g/cm^2^, mean ± SD)	-	−3.06 ± 0.97	-	−2.98 ± 1.26	-	−3.06 ± 0.97	-

Abbreviations: OVCF, osteoporotic vertebral compression fracture; SBP, systolic blood pressure; DBP, diastolic blood pressure; FBS, fasting blood sugar; Hcy, homocysteine; BMI, body mass index; HDL, high density lipoprotein; LDL, low density lipoprotein; TG, triglyceride; DPD, deoxypyridinoline; BMD, bone mineral density.

**Table 2:** Comparison of genotype frequencies and adjusted odds ratio (AOR) values of plasminogen activator inhibitor-1 (*PAI-1*) gene polymorphisms between the osteoporosis and control subjects.

Genotypes	Controls (*n* = 197)	Osteoporosis (*n* = 158)	AOR (95% CI) *	*p*	Non-OVCF (*n* = 71)	AOR (95% CI) *	*p*	OVCF (*n* = 87)	AOR (95% CI) *	*p*
*PAI-1* −844	-	-	-	-	-	-	-	-	-	-
GG	70 (35.5)	40 (25.3)	1.000 (reference)	-	22 (31.0)	1.000 (reference)	-	18 (20.7)	1.000 (reference)	-
GA	91 (46.2)	89 (56.3)	1.584 (0.951–2.640)	0.077	33 (46.5)	1.168 (0.604–2.256)	0.645	56 (64.4)	2.244 (1.164–4.326)	0.016
AA	36 (18.3)	29 (18.4)	1.263 (0.647–2.467)	0.494	16 (22.5)	1.460 (0.641–3.327)	0.368	13 (14.9)	1.052 (0.415–2.664)	0.915
Dominant (GG vs. GA + AA)	-	-	1.496 (0.919–2.435)	0.105	-	1.252 (0.677–2.318)	0.474	-	1.918 (1.016–3.621)	0.044
Recessive (GG + GA vs. AA)	-	-	0.949 (0.528–1.705)	0.860	-	1.342 (0.657–2.740)	0.420	-	0.620 (0.278–1.385)	0.244
HWE-*P*	0.502	0.097	-	-	-	-	-	-	-	-
*PAI-1* −675 4G5G	-	-	-	-	-	-	-	-	-	-
4G4G	113 (57.4)	69 (43.7)	1.000 (reference)		33 (46.5)	1.000 (reference)	-	36 (41.4)	1.000 (reference)	-
4G5G	75 (38.1)	70 (44.3)	1.550 (0.967–2.485)	0.069	32 (45.1)	1.316 (0.716–2.419)	0.376	38 (43.7)	1.653 (0.921–2.968)	0.092
5G5G	9 (4.6)	19 (12.0)	3.032 (1.224–7.512)	0.017	6 (8.5)	1.769 (0.552–5.665)	0.337	13 (14.9)	4.646 (1.625–13.29)	0.004
Dominant (4G4G vs. 4G5G + 5G5G)	-	-	1.727 (1.102–2.706)	0.017	-	1.366 (0.764–2.441)	0.293	-	1.969 (1.130–3.430)	0.017
Recessive (4G4G + 4G5G vs. 5G5G)	-	-	2.510 (1.056–5.968)	0.037	-	1.584 (0.516–4.864)	0.422	-	3.378 (1.301–8.769)	0.012
HWE-*P*	0.435	0.847	-	-	-	-	-	-	-	-
*PAI-1* +43	-	-	-	-	-	-	-	-	-	-
GG	180 (91.4)	132 (83.5)	1.000 (reference)	-	61 (85.9)	1.000 (reference)	-	71 (81.6)	1.000 (reference)	-
GA	16 (8.1)	24 (15.2)	2.006 (0.963–4.175)	0.063	8 (11.3)	1.598 (0.596–4.284)	0.352	16 (18.4)	2.421 (1.057–5.546)	0.037
AA	1 (0.5)	2 (1.3)	3.188 (0.270–37.664)	0.358	2 (2.8)	7.504 (0.561–100.480)	0.128	0 (0.0)	N/A	0.998
Dominant (GG vs. GA + AA)	-	-	2.087 (1.027–4.241)	0.042	-	1.941 (0.775–4.860)	0.157	-	2.292 (1.009–5.206)	0.048
Recessive (GG + GA vs. AA)	-	-	2.972 (0.251–35.212)	0.388	-	7.202 (0.531–97.693)	0.138	-	N/A	0.998
HWE-*P*	0.336	0.454	-	-	-	-	-	-	-	-
*PAI-1* +9785	-	-	-	-	-	-	-	-	-	-
GG	182 (92.4)	148 (93.7)	1.000 (reference)	-	68 (95.8)	1.000 (reference)	-	80 (92.0)	1.000 (reference)	-
GA	14 (7.1)	10 (6.3)	1.072 (0.437–2.633)	0.879	3 (4.2)	0.946 (0.252–3.549)	0.934	7 (8.0)	1.324 (0.477–3.669)	0.590
AA	1 (0.5)	0 (0.0)	N/A	0.995	0 (0.0)	N/A	0.998	0 (0.0)	N/A	0.998
Dominant (GG vs. GA + AA)	-	-	1.005 (0.414–2.440)	0.991	-	0.898 (0.241–3.344)	0.872	-	1.227 (0.447–3.365)	0.691
Recessive (GG + GA vs. AA)	-	-	N/A	0.995	-	N/A	0.998	-	N/A	0.998
HWE-*P*	0.217	0.681	-	-	-	-	-	-	-	-
*PAI-1* +11053	-	-	-	-	-	-	-	-	-	-
TT	54 (27.4)	33 (20.9)	1.000 (reference)		15 (21.1)	1.000 (reference)	-	18 (20.7)	1.000 (reference)	-
TG	101 (51.3)	85 (53.8)	1.347 (0.768–2.362)	0.298	36 (50.7)	1.036 (0.493–2.176)	0.926	49 (56.3)	1.647 (0.814–3.334)	0.166
GG	42 (21.3)	40 (25.3)	1.655 (0.858–3.190)	0.133	20 (28.2)	1.920 (0.825–4.470)	0.130	20 (23.0)	1.521 (0.664–3.483)	0.322
Dominant (TT vs. TG + GG)	-	-	1.429 (0.842–2.428)	0.186	-	1.253 (0.632–2.483)	0.518	-	1.593 (0.814–3.116)	0.174
Recessive (TT + TG vs. GG)	-	-	1.285 (0.762–2.165)	0.347	-	1.499 (0.773–2.907)	0.231	-	1.108 (0.581–2.114)	0.755
HWE-*P*	0.682	0.326	-	-	-	-	-	-	-	-

* The adjusted odds ratio on the basis of risk factors, such as age, hypertension, diabetes mellitus. HWE-*P*: Hardy–Weinberg equilibrium *p*-value. N/A, not applicable.

**Table 3:** Comparison of genotype frequencies of *PAI-1* gene haplotype between the osteoporosis, non-OVCF, OVCF patients and control subjects.

Haplotype	Overall (*n* = 355)	Control (*n* = 197)	Case (*n* = 158)	OR (95% CI)	*p ^a^*	Non-OVCF (*n* = 71)	OR (95% CI)	*p ^a^*	OVCF (*n* = 87)	OR (95% CI)	*p ^a^*
*PAI-1* −844/−675/+43/+9785/+11053			-	-	-	-	-	-	-	-	-
G-4G-G-G-T	0.179	0.237	0.098	1.000 (reference)	-	0.107	1.000 (reference)	-	0.133	1.000 (reference)	-
G-4G-G-G-G	0.092	0.099	0.092	2.231 (1.189–4.187)	0.015	0.127	2.862 (1.311–6.247)	0.013	0.099	1.763 (0.849–3.658)	0.177
G-4G-G-A-T	0.008	0.019	0.000	0.198 (0.011–3.567)	0.197	0.000	0.402 (0.022–7.408)	0.592	0.002	0.265 (0.015–4.816)	0.346
G-4G-G-A-G	0.006	0.007	0.000	0.424 (0.023–8.443)	1.000	0.000	0.862 (0.042–17.52)	1.000	0.015	4.043 (0.765–21.36)	0.110
G-4G-A-G-T	0.007	0.000	0.032	62.33 (3.547–109.5)	<0.0001	0.008	18.10 (0.704–465.0)	0.147	0.012	19.89 (0.923–428.8)	0.044
G-4G-A-G-G	0.021	0.007	0.025	8.000 (1.996–32.06)	0.002	0.000	0.862 (0.042–17.52)	1.000	0.000	0.568 (0.028–11.40)	1.000
G-4G-A-A-T	0.004	0.000	0.004	8.905 (0.353–224.4)	0.256	0.000	N/A	-	0.006	11.94 (0.471–302.7)	0.205
G-5G-G-G-T	0.157	0.158	0.168	2.565 (1.483–4.434)	0.001	0.173	2.500 (1.221–5.118)	0.013	0.140	1.565 (0.812–3.017)	0.184
G-5G-G-G-G	0.029	0.018	0.043	5.571 (2.039–15.22)	0.001	0.047	6.200 (1.903–20.20)	0.004	0.058	5.776 (1.984–16.82)	0.001
G-5G-G-A-T	0.009	0.006	0.000	0.594 (0.028–12.71)	1.000	0.013	6.200 (0.810–47.44)	0.109	0.004	2.022 (0.176–23.29)	0.495
G-5G-G-A-G	0.002	0.003	0.000	0.989 (0.039–24.93)	1.000	0.001	2.011 (0.078–51.66)	1.000	0.001	1.326 (0.052–33.63)	1.000
G-5G-A-G-T	0.042	0.027	0.062	5.455 (2.353–12.64)	<0.0001	0.067	5.636 (2.042–15.56)	0.001	0.061	4.043 (1.560–10.48)	0.006
G-5G-A-G-G	0.007	0.003	0.007	6.000 (0.526–68.51)	0.165	0.001	2.011 (0.078–51.66)	1.000	0.001	1.326 (0.052–33.63)	1.000
G-5G-A-A-T	0.000	0.003	0.000	0.989 (0.039–24.93)	1.000	0.001	2.011 (0.078–51.66)	1.000	0.001	1.326 (0.052–33.63)	1.000
A-4G-G-G-T	0.062	0.076	0.056	1.800 (0.883–3.668)	0.132	0.079	2.273 (0.943–5.483)	0.089	0.028	0.674 (0.236–1.928)	0.621
A-4G-G-G-G	0.310	0.311	0.283	2.195 (1.346–3.580)	0.002	0.361	2.571 (1.361–4.855)	0.004	0.329	1.874 (1.077–3.261)	0.032
A-4G-G-A-T	0.006	0.003	0.005	6.000 (0.526–68.51)	0.165	0.008	6.200 (0.368–104.6)	0.271	0.010	8.087 (0.702–93.16)	0.111
A-4G-A-G-T	0.009	0.003	0.000	0.989 (0.039–24.93)	1.000	0.001	2.011 (0.078–51.66)	1.000	0.001	1.326 (0.052–33.63)	1.000
A-4G-A-G-G	0.014	0.003	0.046	45.00 (5.706–354.9)	<0.0001	0.001	2.011 (0.078–51.66)	1.000	0.001	1.326 (0.052–33.63)	1.000
A-5G-G-G-T	0.011	0.000	0.020	38.59 (2.112–705.1)	0.0004	0.000	N/A	-	0.076	107.4 (6.156–1875)	<0.0001
A-5G-G-G-G	0.011	0.018	0.006	0.857 (0.169–4.347)	1.000	0.000	0.402 (0.022–7.408)	0.592	0.011	1.155 (0.225–5.937)	1.000
A-5G-A-G-T	0.013	0.000	0.029	56.40 (3.188–997.7)	<0.0001	0.010	18.100 (0.704–465.0)	0.147	0.013	19.89 (0.923–428.8)	0.044

*^a^* Fisher’s exact test. N/A, not applicable.

## Supplementary Materials

Supplementary materials can be found at www.mdpi.com/1422-0067/17/12/2062/s1.
