# Supplementary material for: Association of Plasminogen Activator Inhibitor-1 (PAI-1) Gene Polymorphisms with Osteoporotic Vertebral Compression Fractures (OVCFs) in Postmenopausal Women
Source: Int J Mol Sci. 2016 Dec 9;17(12):2062. doi: 10.3390/ijms17122062 (PMC5187862; doi:10.3390/ijms17122062)
Supplement: Supplementary file 1 [file ijms-17-02062-s001.pdf]

Supplementary Materials: Association of Plasminogen Activator Inhibitor-1 (*PAI-1*) Gene Polymorphisms with Osteoporotic Vertebral Compression Fractures (OVCFs) in Postmenopausal Women

Jung Oh Kim, Soo Hong Han, Yeon Ho Lee, Tae Keun Ahn, Jae Joon Lim, Young Sun Chung, Dong Eun Shin, Woo Sik Lee, In Bo Han and Nam Keun Kim

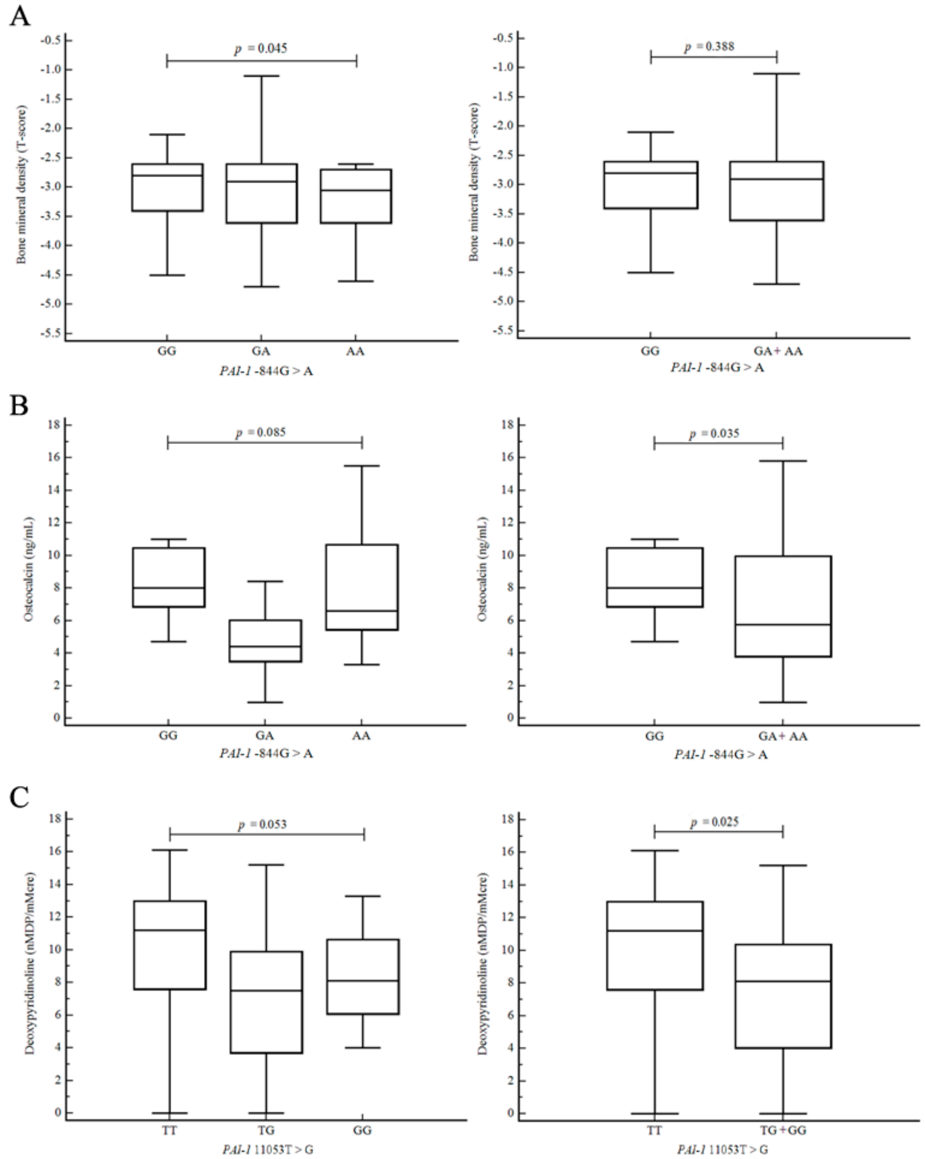

Figure S1. The bone mineral density (BMD), osteocalcin (bone Gla protein, BGP), and deoxypyridinoline (DPD) levels according to *PAI-1* gene polymorphisms in OVCF patients. The statistical analysis was performed by Kruskal-Wallis or Mann-Whitney test for *PAI-1* −844G > A, 11053T > G genotype, respectively. The black line in box was mean of factor value. (**A**) The BMD level: The BMD (*T*-score) of lumbar spine (L2–L4) is significantly different (p = 0.045) between PAI-1 −844GG (−3.19 ± 0.84), GA (−2.90 ± 1.01) and AA genotype (−3.44 ± 0.94); (**B**) plasma BGP level: we found that *PAI-1* −844G > A polymorphism is making a difference with plasma BGP level. The *PAI-1* −844GA + AA genotype had a descend osteocalcin level (6.77 ± 3.99) compared with the −844GG genotype (11.78 ± 10.67); (**C**) plasma DPD level: the *PAI-1* 11053TG + GG genotype had a descend DPD level (7.14 ± 4.18) compared with the 11053TT genotype (12.15 ± 7.79).

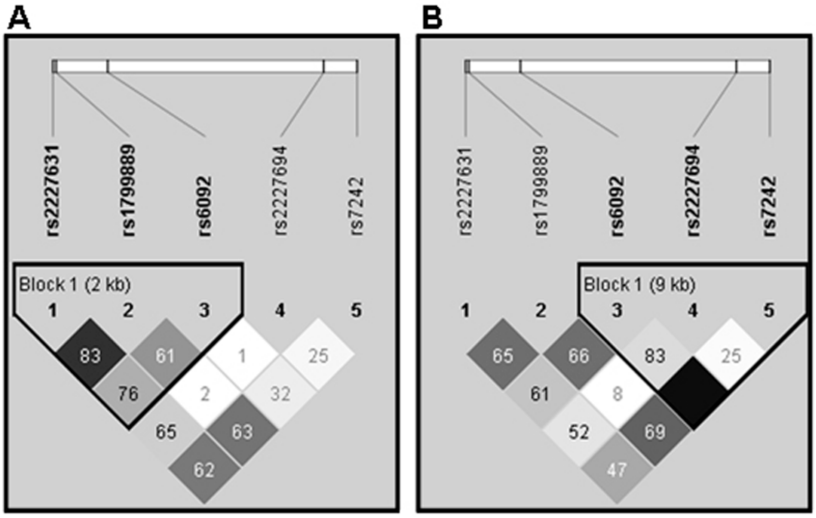

Figure S2. Linkage disequilibrium (LD) patterns of *PAI-1* gene SNPs. Values in squares represent the LD between single markers. The color of area were show "Linkage disequilibrium", that high association with two loci as it gets darker. (**A**) Control individuals exhibited strong linkage disequilibrium between loci −844 and −675 (D′ = 0.838, LOD = 8.42, r^2 = 0.153), −844 and +43 (D′ = 0.764, LOD = 1.2, r^2 = 0.02); (**B**) whereas osteoporosis patients exhibited strong linkage disequilibrium between loci +43 and +11053 (D′ = 1.000, LOD = 4.51, r^2 = 0.106), +43 and +9785 (D′ = 0.831, LOD = 0.06, r^2 = 0.002).

Table S1. Baseline characteristics between OVCF and non-OVCF patients.

Characteristic	OVCF (*n* = 87)	non-OVCF (*n* = 71)	*p*
Age (years, mean ± SD)	70.23 ± 9.44	70.83 ± 6.53	0.846
Hypertension (%)	37 (42.5)	20 (28.2)	0.01
SBP (mmHg, mean ± SD)	129.99 ± 16.51	126.34 ± 13.89	0.183
DBP (mmHg, mean ± SD)	77.63 ± 10.57	74.72 ± 10.26	0.062
Diabetes mellitus (%)	11 (12.6)	17 (23.9)	0.58
FBS (mg/dL, mean ± SD)	135.89 ± 53.96	114.30 ± 26.15	0.003
Hcy (μmol/L, mean ± SD)	10.01 ± 3.85	9.57 ± 4.31	0.499
Folate (ng/mL, mean ± SD)	6.54 ± 4.03	10.19 ± 4.91	<0.0001
BMI (kg/m^2, mean ± SD)	23.87 ± 5.02	23.77 ± 2.79	0.222
HDL-cholesterol (mg/dL, mean ± SD)	44.39 ± 15.95	44.83 ± 12.24	0.868
LDL-cholesterol (mg/dL, mean ± SD)	117.87 ± 42.82	95.02 ± 32.02	0.002
TG (mg/dL, mean ± SD)	147.62 ± 74.17	132.23 ± 86.68	0.285
Vitamin B12 (pg/mL, mean ± SD)	973.11 ± 1024.37	639.46 ± 384.93	0.028
25-(OH) Vitamin D (ng/mL, mean ± SD)	41.97 ± 34.20	18.57 ± 11.62	<0.0001
Osteocalcin (ng/mL, mean ± SD)	7.83 ± 6.39	-	-
Deoxypyridinoline (nMDP/mMcre, mean ± SD)	8.41 ± 5.55	-	-
BMD (g/cm^2, mean ± SD)	−2.98 ± 1.26	−3.06 ± 0.97	0.394

Abbreviations: OVCF, osteoporotic vertebral compression fracture; SBP, systolic blood pressure; DBP, diastolic blood pressure; FBS, fasting blood sugar; Hcy, homocysteine; BMI, body mass index; HDL, high density lipoprotein; LDL, low density lipoprotein; TG, triglyceride; BMD, bone mineral density.

Table S2. Comparison of genotype frequencies and AOR values of *PAI-1* polymorphisms according to with/without fracture patients.

Genotypes	non-OVCF (*n* = 71)	OVCF (*n* = 87)	AOR (95% CI) *	*p*
PAI-1 −844	-	-	-	-
GG	22 (31.0)	18 (20.7)	1.000 (reference)	-
GA	33 (46.5)	56 (64.4)	2.456 (1.063–5.676)	0.036
AA	16 (22.5)	13 (14.9)	0.862 (0.274–2.716)	0.800
Dominant (GG vs. GA + AA)	-	-	1.884 (0.848–4.183)	0.120
Recessive (GG + GA vs. AA)	-	-	0.431 (0.170–1.094)	0.077
PAI-1 −675 4G5G	-	-	-	-
4G4G	33 (46.5)	36 (41.4)	-	-
4G5G	32 (45.1)	38 (43.7)	1.084 (0.527–2.230)	0.827
5G5G	6 (8.5)	13 (14.9)	1.578 (0.490–5.082)	0.444
Dominant (4G4G vs. 4G5G + 5G5G)	-	-	1.207 (0.610–2.389)	0.589
Recessive (4G4G + 4G5G vs. 5G5G)	-	-	1.614 (0.535–4.869)	0.395
PAI-1 +43	-	-	-	-
GG	61 (85.9)	71 (81.6)	-	-
GA	8 (11.3)	16 (18.4)	1.599 (0.589–4.341)	0.357
AA	2 (2.8)	0 (0.0)	N/A	0.998
Dominant (GG vs. GA + AA)	-	-	1.286 (0.500–3.303)	0.602
Recessive (GG + GA vs. AA)	-	-	N/A	0.998
PAI-1 +9785	-	-	-	-
GG	68 (95.8)	80 (92.0)	-	-
GA	3 (4.2)	7 (8.0)	1.675 (0.385–7.293)	0.492
AA	0 (0.0)	0 (0.0)	N/A	-
Dominant (GG vs. GA + AA)	-	-	1.675 (0.385–7.293)	0.492
Recessive (GG + GA vs. AA)	-	-	N/A	-
PAI-1 +11053	-	-	-	-
TT	15 (21.1)	18 (20.7)	-	-
TG	36 (50.7)	49 (56.3)	1.316 (0.534–3.246)	0.551
GG	20 (28.2)	20 (23.0)	0.916 (0.326–2.575)	0.868
Dominant (TT vs. TG + GG)	-	-	1.148 (0.494–2.666)	0.748
Recessive (TT + TG vs. GG)	-	-	0.803 (0.373–1.728)	0.575

* The adjusted odds ratio on the basis of risk factors, such as age, hypertension, diabetes mellitus. Abbreviations: AOR, adjusted odds ratio; N/A, not applicable.

Table S3. Comparison of genotype frequencies of the *PAI-1* gene 4 site haplotype in the context of OVCF in postmenopausal women and control subjects.

Haplotype	Overall (2*n* = 710)	Control (2*n* = 394)	Case (2*n* = 316)	OR (95% CI)	*p* [a]
	PAI-1 −844/−675/+43/+9785		-	-	-
G-4G-G-G	0.274	0.332	0.194	1.000 (reference)	-
G-4G-G-A	0.011	0.024	0.000	0.102 (0.006–1.767)	0.034
G-4G-A-G	0.028	0.010	0.052	9.127 (2.945–28.28)	<0.0001
G-4G-A-A	0.005	0.000	0.008	10.69 (0.505–226.2)	0.104
G-5G-G-G	0.189	0.180	0.208	1.996 (1.270–3.139)	0.003
G-5G-G-A	0.008	0.008	0.000	0.306 (0.016–6.010)	0.553
G-5G-A-G	0.049	0.029	0.074	4.116 (1.922–8.814)	0.000
G-5G-A-A	0.000	0.003	0.000	0.713 (0.029–17.76)	1.000
A-4G-G-G	0.366	0.388	0.339	1.502 (1.015–2.222)	0.049
A-4G-G-A	0.011	0.006	0.021	7.516 (1.516–37.26)	0.008
A-4G-A-G	0.022	0.004	0.045	15.03 (3.312–68.24)	<0.0001
A-5G-G-G	0.021	0.016	0.027	2.863 (0.952–8.615)	0.076
A-5G-A-G	0.015	0.000	0.031	44.90 (2.587–779.3)	<0.0001
A-5G-A-A	0.001	0.000	0.003	6.415 (0.257–159.9)	0.321
	PAI-1 −844/−675/+43/+11053		-	-	-
G-4G-G-T	0.187	0.256	0.100	1.000 (reference)	-
G-4G-G-G	0.098	0.106	0.093	2.179 (1.174–4.044)	0.016
G-4G-A-T	0.015	0.000	0.035	71.83 (4.115–1254)	<0.0001
G-4G-A-G	0.016	0.006	0.025	8.417 (2.106–33.64)	0.002
G-5G-G-T	0.166	0.163	0.169	2.614 (1.524–4.482)	0.001
G-5G-G-G	0.031	0.022	0.039	4.208 (1.625–10.90)	0.004
G-5G-A-T	0.041	0.030	0.061	4.997 (2.190–11.40)	0.0002
G-5G-A-G	0.009	0.003	0.014	12.63 (1.361–117.1)	0.017
A-4G-G-T	0.069	0.079	0.061	1.934 (0.965–3.880)	0.067
A-4G-G-G	0.308	0.311	0.300	2.458 (1.521–3.971)	0.0002
A-4G-A-T	0.003	0.003	0.000	1.041 (0.041–26.20)	1.000
A-4G-A-G	0.020	0.003	0.045	44.19 (5.588–349.4)	<0.0001
A-5G-G-T	0.010	0.000	0.020	40.60 (2.225–740.9)	0.0000
A-5G-G-G	0.011	0.018	0.006	0.902 (0.178–4.563)	1.000
A-5G-A-T	0.015	0.000	0.033	65.58 (3.737–1151)	<0.0001
	PAI-1 −844/−675/+9785/+11053		-	-	-
G-4G-G-T	0.190	0.235	0.132	1.000 (reference)	-
G-4G-G-G	0.108	0.107	0.121	2.003 (1.132–3.544)	0.020
G-4G-A-T	0.012	0.019	0.000	0.147 (0.008–2.629)	0.105
G-4G-A-G	0.007	0.007	0.002	0.314 (0.016–6.225)	0.553
G-5G-G-T	0.197	0.185	0.228	2.184 (1.340–3.560)	0.002
G-5G-G-G	0.039	0.021	0.049	4.429 (1.758–11.16)	0.002
G-5G-A-T	0.010	0.009	0.000	0.314 (0.016–6.225)	0.553
G-5G-A-G	0.003	0.003	0.003	2.214 (0.135–36.28)	0.531
A-4G-G-T	0.067	0.080	0.056	1.286 (0.648–2.553)	0.482
A-4G-G-G	0.329	0.313	0.324	1.836 (1.172–2.877)	0.008
A-4G-A-T	0.006	0.003	0.007	4.429 (0.370–50.23)	0.238
A-4G-A-G	0.000	0.000	0.016	24.20 (1.307–448.0)	0.004
A-5G-G-T	0.027	0.000	0.051	72.60 (4.252–1239)	<0.0001
A-5G-G-G	0.009	0.018	0.007	0.633 (0.126–3.176)	0.722
A-5G-A-T	0.000	0.000	0.004	6.600 (0.263–165.5)	0.316

Table S3. *Cont.*

Haplotype	Overall (2*n* = 710)	Control (2*n* = 394)	Case (2*n* = 316)	OR (95% CI)	*p* [a]
	PAI-1 −844/+43/+9785/+11053		-	-	-
G-G-G-T	0.339	0.391	0.266	1.000 (reference)	-
G-G-G-G	0.122	0.118	0.135	1.677 (1.026–2.743)	0.043
G-G-A-T	0.017	0.025	0.000	0.087 (0.005–1.505)	0.018
G-G-A-G	0.007	0.010	0.000	0.203 (0.011–3.822)	0.301
G-A-G-T	0.047	0.029	0.090	4.667 (2.212–9.846)	<0.0001
G-A-G-G	0.027	0.011	0.041	5.958 (1.883–18.86)	0.001
G-A-A-T	0.005	0.003	0.000	0.610 (0.025–15.14)	1.000
G-A-A-G	0.002	0.000	0.004	5.485 (0.221–136.2)	0.356
A-G-G-T	0.074	0.080	0.077	1.375 (0.760–2.486)	0.355
A-G-G-G	0.316	0.327	0.287	1.293 (0.886–1.887)	0.211
A-G-A-T	0.005	0.003	0.004	1.833 (0.113–29.71)	1.000
A-G-A-G	0.002	0.000	0.019	23.77 (1.322–427.4)	0.002
A-A-G-T	0.021	0.000	0.037	45.71 (2.671–782.2)	<0.0001
A-A-G-G	0.019	0.004	0.037	22.00 (2.810–172.2)	<0.0001
A-A-A-T	0.000	0.000	0.005	5.485 (0.221–136.2)	0.356
	PAI-1 −675/+43/+9785/+11053		-	-	-
4G-G-G-T	0.243	0.313	0.155	1.000 (reference)	-
4G-G-G-G	0.393	0.408	0.373	1.840 (1.224–2.766)	0.004
4G-G-A-T	0.013	0.018	0.001	0.166 (0.009–2.970)	0.192
4G-G-A-G	0.012	0.012	0.020	3.012 (0.878–10.33)	0.090
4G-A-G-T	0.017	0.004	0.036	27.61 (3.470–219.8)	<0.0001
4G-A-G-G	0.037	0.010	0.070	13.81 (4.523–42.14)	<0.0001
4G-A-A-T	0.002	0.000	0.004	7.485 (0.300–187.0)	0.289
5G-G-G-T	0.170	0.161	0.189	2.391 (1.473–3.881)	0.001
5G-G-G-G	0.043	0.035	0.050	2.896 (1.302–6.322)	0.011
5G-G-A-T	0.005	0.006	0.000	0.499 (0.024–10.59)	1.000
5G-G-A-G	0.001	0.003	0.000	0.832 (0.033–20.78)	1.000
5G-A-G-T	0.055	0.027	0.090	6.390 (2.952–13.83)	<0.0001
5G-A-G-G	0.006	0.003	0.006	5.020 (0.445–56.67)	0.204
5G-A-A-T	0.003	0.003	0.003	2.510 (0.154–40.96)	0.493
5G-A-A-G	0.001	0.000	0.003	7.485 (0.300–187.0)	0.289

[a] Fisher's exact test.

Table S4. Comparison of genotype frequencies of the *PAI-1* gene 3 site haplotype in the context of OVCF in postmenopausal women and control subjects.

Haplotype	Overall (2*n* = 710)	Control (2*n* = 394)	Case (2*n* = 316)	OR (95% CI)	*p* [a]
PAI-1 −844/−675/+43	-	-	-	-	-
G-4G-G	0.287	0.356	0.196	1.000 (reference)	-
G-4G-A	0.032	0.010	0.059	10.73 (3.502–32.85)	<0.0001
G-5G-G	0.197	0.188	0.207	2.014 (1.288–3.148)	0.002
G-5G-A	0.048	0.032	0.072	3.995 (1.900–8.399)	0.0002
A-4G-G	0.376	0.394	0.358	1.646 (1.120–2.419)	0.012
A-4G-A	0.022	0.004	0.045	15.81 (3.485–71.68)	<0.0001
A-5G-G	0.021	0.016	0.027	3.387 (1.155–9.931)	0.042
A-5G-A	0.017	0.000	0.035	51.70 (2.997–891.9)	<0.0001
PAI-1 −844/−675/+9785	-	-	-	-	-
G-4G-G	0.303	0.342	0.252	1.000 (reference)	-
G-4G-A	0.016	0.024	0.006	0.375 (0.079–1.780)	0.335
G-5G-G	0.235	0.209	0.275	1.790 (1.189–2.696)	0.007
G-5G-A	0.009	0.011	0.002	0.422 (0.046–3.843)	0.654
A-4G-G	0.387	0.392	0.381	1.315 (0.913–1.895)	0.165
A-4G-A	0.011	0.006	0.020	5.063 (0.994–25.69)	0.057
A-5G-G	0.039	0.016	0.060	5.344 (2.048–13.94)	0.0002
A-5G-A	0.001	0.000	0.004	5.050 (0.203–125.5)	0.375
PAI-1 −844/−675/+11053	-	-	-	-	-
G-4G-T	0.202	0.254	0.133	1.000 (reference)	-
G-4G-G	0.114	0.114	0.121	2.011 (1.146–3.529)	0.021
G-5G-T	0.206	0.194	0.228	2.256 (1.391–3.659)	0.001
G-5G-G	0.041	0.024	0.053	4.048 (1.712–9.569)	0.002
A-4G-T	0.072	0.083	0.062	1.443 (0.744–2.798)	0.302
A-4G-G	0.329	0.313	0.342	2.091 (1.342–3.258)	0.001
A-5G-T	0.027	0.000	0.054	82.76 (4.862–1409)	<0.0001
A-5G-G	0.009	0.018	0.007	0.680 (0.136–3.412)	1.000
PAI-1 −844/+43/+9785	-	-	-	-	-
G-G-G	0.464	0.513	0.402	1.000 (reference)	-
G-G-A	0.020	0.032	0.000	0.059 (0.003–0.999)	0.003
G-A-G	0.074	0.039	0.123	4.135 (2.190–7.809)	<0.0001
G-A-A	0.006	0.003	0.010	4.772 (0.491–46.40)	0.303
A-G-G	0.386	0.403	0.365	1.150 (0.830–1.595)	0.406
A-G-A	0.011	0.006	0.022	5.567 (1.138–27.23)	0.032
A-A-G	0.040	0.005	0.079	19.88 (4.628–85.41)	<0.0001
PAI-1 −844/+43/+9785	-	-	-	-	-
G-G-T	0.355	0.416	0.269	1.000 (reference)	-
G-G-G	0.129	0.128	0.132	1.589 (0.978–2.581)	0.078
G-A-T	0.052	0.031	0.084	4.341 (2.094–8.988)	<0.0001
G-A-G	0.028	0.010	0.049	7.718 (2.501–23.82)	0.0002
A-G-T	0.079	0.083	0.080	1.462 (0.817–2.616)	0.225
A-G-G	0.318	0.327	0.307	1.451 (1.001–2.104)	0.059
A-A-T	0.021	0.000	0.045	55.80 (3.286–947.3)	<0.0001
A-A-G	0.019	0.004	0.034	21.22 (2.693–167.2)	<0.0001
PAI-1 −844/+9785/+11053	-	-	-	-	-
G-G-T	0.388	0.420	0.357	1.000 (reference)	-
G-G-G	0.146	0.129	0.172	1.546 (0.984–2.428)	0.065
G-A-T	0.021	0.028	0.000	0.063 (0.004–1.087)	0.004
G-A-G	0.009	0.010	0.006	0.730 (0.131–4.055)	1.000
A-G-T	0.092	0.080	0.109	1.551 (0.905–2.659)	0.128
A-G-G	0.338	0.331	0.331	1.179 (0.830–1.676)	0.371
A-A-T	0.007	0.003	0.012	5.841 (0.644–52.97)	0.164
A-A-G	0.000	0.000	0.013	13.12 (0.699–246.3)	0.029

Table S4. *Cont.*

Haplotype	Overall (2*n* = 710)	Control (2*n* = 394)	Case (2*n* = 316)	OR (95% CI)	*p* [a]
PAI-1 −675/+43/+9785		-	-	-	-
4G-G-G	0.637	0.720	0.529	1.000 (reference)	-
4G-G-A	0.026	0.031	0.022	0.992 (0.383–2.570)	1.000
4G-A-G	0.052	0.014	0.102	10.88 (4.159–28.48)	<0.0001
4G-A-A	0.002	0.000	0.005	8.493 (0.401–178.1)	0.139
5G-G-G	0.212	0.197	0.237	1.656 (1.143–2.401)	0.010
5G-G-A	0.005	0.007	0.000	0.243 (0.012–4.730)	0.301
5G-A-G	0.062	0.030	0.100	4.535 (2.273–9.047)	<0.0001
5G-A-A	0.003	0.003	0.005	1.701 (0.106–27.39)	1.000
PAI-1 −675/+9785/+11053		-	-	-	-
4G-G-T	0.257	0.331	0.159	1.000 (reference)	-
4G-G-G	0.405	0.419	0.394	1.969 (1.319–2.939)	0.001
4G-A-T	0.018	0.004	0.038	31.44 (3.982–248.3)	<0.0001
4G-A-G	0.037	0.010	0.068	13.76 (4.497–42.08)	<0.0001
5G-G-T	0.176	0.166	0.189	2.418 (1.498–3.904)	0.0002
5G-G-G	0.042	0.038	0.046	2.620 (1.193–5.753)	0.019
5G-A-T	0.057	0.030	0.092	6.332 (2.998–13.37)	<0.0001
5G-A-G	0.009	0.003	0.014	10.48 (1.143–96.10)	0.026
PAI-1 +43/+9785/+11053		-	-	-	-
G-G-T	0.417	0.476	0.348	1.000 (reference)	-
G-G-G	0.432	0.440	0.418	1.304 (0.941–1.808)	0.115
G-A-T	0.017	0.023	0.000	0.090 (0.005–1.559)	0.029
G-A-G	0.014	0.015	0.022	1.994 (0.653–6.085)	0.249
A-G-T	0.069	0.028	0.124	6.060 (2.981–12.32)	<0.0001
A-G-G	0.045	0.015	0.078	7.121 (2.833–17.90)	<0.0001
A-A-T	0.004	0.003	0.005	3.418 (0.306–38.16)	0.558
A-A-G	0.002	0.000	0.005	8.529 (0.406–179.4)	0.139

[a] Fisher's exact test.

Table S5. Comparison of genotype frequencies of the *PAI-1* gene 2 site haplotype in the context OVCF in postmenopausal women and control subjects.

Haplotype	Overall (2*n* = 710)	Control (2*n* = 394)	Case (2*n* = 316)	OR (95% CI)	*p* [a]
PAI-1 −844/−675		-	-	-	-
G-4G	0.319	0.366	0.258	1.000 (reference)	-
G-5G	0.244	0.22	0.277	1.798 (1.202–2.689)	0.004
A-4G	0.398	0.398	0.401	1.438 (1.004–2.059)	0.057
A-5G	0.039	0.016	0.065	5.926 (2.287–15.36)	<0.0001
PAI-1 −844/+43		-	-	-	-
G-G	0.483	0.545	0.402	1.000 (reference)	-
G-A	0.08	0.041	0.133	4.444 (2.399–8.231)	<0.0001
A-G	0.397	0.409	0.386	1.283 (0.930–1.770)	0.14
A-A	0.039	0.005	0.079	21.16 (4.928–90.87)	<0.0001
PAI-1 −844/+9785		-	-	-	-
G-G	0.538	0.552	0.527	1.000 (reference)	-
G-A	0.025	0.035	0.008	0.280 (0.079–0.991)	0.044
A-G	0.425	0.408	0.442	1.137 (0.839–1.540)	0.439
A-A	0.011	0.006	0.024	4.575 (0.938–22.32)	0.048
PAI-1 −844/+11053		-	-	-	-
G-T	0.409	0.448	0.359	1.000 (reference)	-
G-G	0.154	0.139	0.175	1.544 (0.992–2.402)	0.055
A-T	0.098	0.083	0.119	1.731 (1.024–2.927)	0.044
A-G	0.339	0.331	0.347	1.306 (0.924–1.847)	0.134
PAI-1 −675/+43		-	-	-	-
4G-G	0.663	0.75	0.552	1.000 (reference)	-
4G-A	0.054	0.014	0.106	11.57 (4.440–30.14)	<0.0001
5G-G	0.218	0.204	0.236	1.595 (1.105–2.301)	0.014
5G-A	0.066	0.032	0.106	4.318 (2.213–8.428)	<0.0001
PAI-1 −675/+9785		-	-	-	-
4G-G	0.69	0.734	0.635	1.000 (reference)	-
4G-A	0.027	0.03	0.023	0.839 (0.325–2.168)	0.815
5G-G	0.274	0.226	0.334	1.696 (1.213–2.371)	0.002
5G-A	0.01	0.011	0.008	1.078 (0.239–4.872)	1
PAI-1 −675/+11053		-	-	-	-
4G-T	0.274	0.334	0.197	1.000 (reference)	-
4G-G	0.443	0.43	0.462	1.839 (1.265–2.675)	0.002
5G-T	0.233	0.196	0.281	2.461 (1.602–3.780)	<0.0001
5G-G	0.05	0.04	0.061	2.528 (1.218–5.249)	0.013
PAI-1 +43/+9785		-	-	-	-
G-G	0.85	0.916	0.767	1.000 (reference)	-
G-A	0.031	0.038	0.021	0.696 (0.280–1.733)	0.511
A-G	0.114	0.043	0.202	5.616 (3.211–9.823)	<0.0001
A-A	0.006	0.003	0.011	4.475 (0.463–43.30)	0.308
PAI-1 +43/+11053		-	-	-	-
G-T	0.434	0.499	0.348	1.000 (reference)	-
G-G	0.446	0.455	0.44	1.391 (1.008–1.919)	0.05
A-T	0.073	0.031	0.13	6.119 (3.086–12.13)	<0.0001
A-G	0.047	0.014	0.082	7.761 (3.099–19.44)	<0.0001
PAI-1 +9785/+11053		-	-	-	-
G-T	0.486	0.504	0.466	1.000 (reference)	-
G-G	0.478	0.455	0.502	1.202 (0.889–1.626)	0.249
A-T	0.021	0.026	0.012	0.542 (0.167–1.761)	0.411
A-G	0.015	0.014	0.02	1.354 (0.428–4.283)	0.768

[a] Fisher's exact test.

Table S6. Stratified analysis of *PAI-1* polymorphisms for hypertension, diabetes mellitus, 25-OH vit. D, vit. B_{12}, folate, and homocysteine levels.

Characteristics	***PAI-1* −844 GA + AA**		***PAI-1* −675 5G5G**		***PAI-1* 43 GA + AA**		***PAI-1* 9785 GA + AA**		***PAI-1* 11053 TG + GG**	
	**AOR (95% CI) ***	***p***	**AOR (95% CI) ***	***p***	**AOR (95% CI) ***	***p***	**AOR (95% CI) ***	***p***	**AOR (95% CI) ***	***p***
Hypertension (−)	2.057 (1.053–4.019)	0.035	1.156 (0.326–4.098)	0.822	2.664 (0.977–7.260)	0.056	0.708 (0.196–2.563)	0.599	1.127 (0.564–2.254)	0.735
Hypertension (+)	1.097 (0.528–2.276)	0.804	8.013 (1.906–33.69)	0.005	1.555 (0.550–4.398)	0.405	1.467 (0.428–5.036)	0.542	1.858 (0.803–4.299)	0.148
Diabetes mellitus (−)	1.721 (1.010–2.932)	0.046	5.058 (1.655–15.46)	0.005	2.050 (0.954–4.406)	0.066	0.916 (0.364–2.304)	0.852	1.303 (0.745–2.280)	0.354
Diabetes mellitus (+)	0.883 (0.231–3.374)	0.856	0.589 (0.082–4.214)	0.598	1.620 (0.217–12.090)	0.638	8.208 (0.264–255.001)	0.23	2.849 (0.502–16.185)	0.237
25-OH vit. D ≥ 30 ng/mL	0.440 (0.108–1.791)	0.252	19.126 (0.998–366.8)	0.05	2.910 (0.510–16.618)	0.23	N/A	0.998	0.664 (0.148–2.978)	0.592
25-OH vit. D < 30 ng/mL	1.643 (0.780–3.461)	0.192	1.208 (0.200–7.306)	0.837	0.853 (0.272–2.680)	0.786	0.825 (0.169–4.020)	0.812	1.245 (0.563–2.751)	0.589
Vit. B_{12} > 400 pg/mL	1.314 (0.650–2.654)	0.447	1.800 (0.403–8.039)	0.441	1.968 (0.772–5.017)	0.156	0.339 (0.042–2.737)	0.31	1.113 (0.519–2.387)	0.784
Vit. B_{12} ≤ 400 pg/mL	1.042 (0.410–2.652)	0.931	3.604 (0.590–22.04)	0.165	1.249 (0.309–5.039)	0.755	1.930 (0.383–9.727)	0.425	1.187 (0.403–3.490)	0.756
Folate > 4.40 nmol/L [a]	1.774 (1.016–3.096)	0.044	2.397 (0.876–6.558)	0.089	1.737 (0.802–3.762)	0.162	0.706 (0.243–2.050)	0.522	1.476 (0.818–2.665)	0.196
Folate ≤ 4.40 nmol/L [a]	1.230 (0.310–4.885)	0.768	N/A	N/A	7.406 (0.716–76.644)	0.093	N/A	N/A	0.933 (0.207–4.210)	0.928
tHcy < 12.41 μmol/L [b]	1.512 (0.393–5.822)	0.548	5.543 (0.566–54.29)	0.141	1.812 (0.842–3.900)	0.128	1.794 (0.145–22.18)	0.649	0.670 (0.150–2.991)	0.599
tHcy ≥ 12.41 μmol/L [b]	1.590 (0.929–2.720)	0.091	2.394 (0.839–6.832)	0.103	5.133 (0.513–51.334)	0.164	0.860 (0.312–2.369)	0.77	1.581 (0.884–2.829)	0.123

* 95% CI indicates 95% confidence interval. Adjusted by age, hypertension, diabetes mellitus; [a] 4.40 nmol/L is based on the bottom 15% of the folate level in patients and control group; [b] 12.41 μmol/L is based on the top. Abbreviations: AOR, adjusted odds ratio; N/A, not applicable.

Table S7. Combined effects of *PAI-1* genotype and homocysteine and folate levels on OVCF risk in postmenopausal women.

Genotypes	Folate > 4.40 nmol/L [a]	Folate ≤ 4.40 nmol/L [a]	tHcy < 12.41 μmol/L [b]	tHcy ≥ 12.41 μmol/L [b]
PAI-1 −844 GG	1.000 (reference)	3.091 (0.903–10.580)	1.000 (reference)	1.972 (0.587–6.628)
PAI-1 −844 GA + AA	1.774 (1.016–3.096)	2.784 (1.172–6.613)	1.590 (0.929–2.720)	2.522 (1.074–5.921)
PAI-1 −675 4G4G	1.000 (reference)	1.627 (0.591–4.481)	1.000 (reference)	1.394 (0.590–3.295)
PAI-1 −675 4G5G + 5G5G	1.615 (0.981–2.660)	3.625 (1.404–9.362)	1.631 (0.994–2.674)	3.571 (1.212–10.52)
PAI-1 43 GG	1.000 (reference)	1.456 (0.716–2.960)	1.000 (reference)	1.812 (0.842–3.900)
PAI-1 43 GA + AA	1.737 (0.802–3.762)	9.247 (1.049–81.51)	1.450 (0.741–2.834)	8.000 (0.865–73.99)
PAI-1 9785 GG	1.000 (reference)	1.617 (0.816–3.203)	1.000 (reference)	1.608 (0.835–3.095)
PAI-1 9785 GA + AA	0.706 (0.243–2.050)	N/A	0.860 (0.312–2.369)	2.780 (0.237–32.58)
PAI-1 11053 TT	1.000 (reference)	2.607 (0.591–11.49)	1.000 (reference)	3.598 (0.873–14.83)
PAI-1 11053 TG + GG	1.476 (0.818–2.665)	2.468 (1.035–5.887)	1.581 (0.884–2.829)	2.275 (0.952–5.440)

[a] 4.40 nmol/L is based on the bottom 15% folate level in patients and control group; [b] 12.41 μmol/L is based on the top 15% homocysteine level in patients and control group. Abbreviations: N/A, not applicable.

Table S8. The BMD, vit. B_{12}, 25-OH vit. D, osteocalcin, and DPD levels according to *PAI-1* gene polymorphisms in OVCF patients.

Genotypes	BMD (g/cm2)		Vit. B_{12} (pmol/L)		25-Vit. D (ng/mL)		Osteocalcin (ng/mL)		DPD (nMDP/mMcre)	
	**Mean ± SD**	**CV (%)**	**Mean ± SD**	**CV (%)**	**Mean ± SD**	**CV (%)**	**Mean ± SD**	**CV (%)**	**Mean ± SD**	**CV (%)**
PAI-1 −844	-	-	-	-	-	-	-	-	-	-
GG	−3.19 ± 0.84	26.3	720.12 ± 668.90	92.9	28.23 ± 26.10	92.5	11.78 ± 10.67	90.6	7.15 ± 5.14	71.9
GA	−2.90 ± 1.01	34.8	798.06 ± 894.35	112.1	25.42 ± 21.08	82.9	6.34 ± 3.91	61.7	7.68 ± 4.59	59.8
AA	−3.44 ± 0.94	27.3	717.77 ± 368.68	51.4	14.67 ± 11.65	79.4	8.27 ± 4.19	50.7	11.92 ± 8.14	68.3
p	0.045	-	0.688	-	0.15	-	0.085	-	0.207	-
GG vs. GA + AA	−3.02 ± 1.01	33.4	774.47 ± 777.35	100.4	22.32 ± 19.37	86.8	6.77 ± 3.99	58.9	8.62 ± 5.67	65.8
p	0.388	-	0.571	-	0.291	-	0.035 *	-	0.516	-
PAI-1 −675	-	-	-	-	-	-	-	-	-	-
4G4G	−3.03 ± 0.84	27.7	707.41 ± 477.10	67.4	20.86 ± 22.43	107.5	10.30 ± 8.96	87	7.50 ± 4.44	59.2
4G5G	−3.12 ± 1.08	34.6	787.06 ± 861.90	109.5	25.88 ± 21.17	81.8	6.59 ± 3.56	54	7.62 ± 4.91	64.4
5G5G	−2.95 ± 0.97	32.9	1041.54 ± 1634.34	156.9	32.83 ± 16.14	49.2	6.05 ± 3.69	61	13.85 ± 9.11	65.8
p	0.772	-	0.254	-	0.406	-	0.177	-	0.098	-
4G4G vs. 4G5G + 5G5G	−3.09 ± 1.05	34.0	814.18 ± 966.52	118.7	26.77 ± 20.55	76.8	6.46 ± 3.53	54.6	8.75 ± 6.12	69.9
p	0.72	-	0.241	-	0.244	-	0.062	-	0.525	-
PAI-1 +43	-	-	-	-	-	-	-	-	-	-
GG	−3.07 ± 0.99	32.2	761.07 ± 772.88	101.6	23.47 ± 21.97	93.6	7.98 ± 6.73	84.3	8.42 ± 4.01	47.6
GA	−3.10 ± 0.90	29.0	745.63 ± 362.05	48.6	28.93 ± 20.88	72.2	7.34 ± 2.19	29.8	7.50 ± 11.88	158.4
AA	−2.50 ± 0.00	0.0	433.33 ± 221.67	51.2	21.58 ± 9.79	45.4	-	-	-	-
p	0.709	-	0.748	-	0.79	-	0.835	-	0.736	-
GG vs. GA + AA	−3.04 ± 0.88	28.9	710.93 ± 360.20	50.7	27.46 ± 18.96	69	7.34 ± 2.19	29.8	7.50 ± 11.88	158.4
p	0.906	-	0.74	-	0.589	-	0.835	-	0.736	-
PAI-1 +9785	-	-	-	-	-	-	-	-	-	-
GG	−3.05 ± 0.92	30.2	762.75 ± 763.15	100.1	24.39 ± 21.69	88.9	7.83 ± 6.30	80.5	8.28 ± 5.67	68.5
GA	−3.24 ± 1.58	48.8	664.53 ± 317.31	47.7	10.80 ± 0.01	0.1	9.20 ± 9.33	101.4	8.30 ± 2.26	27.2
AA	-	-	627	-	-	-	-	-	-	-
p	0.564	-	0.857	-	0.382	-	0.77	-	0.997	-
GG vs. GA + AA	−3.24 ± 1.58	48.8	662.44 ± 307.96	46.5	10.80 ± 0.01	0.1	9.20 ± 9.33	101.4	8.30 ± 2.26	27.2
p	0.564	-	0.58		0.382	-	0.77	-	0.997	-

Table S8. *Cont.*

Genotypes	BMD (g/cm2)		Vit. B12 (pmol/L)		25-Vit. D (ng/mL)		Osteocalcin (ng/mL)		DPD (nMDP/mMcre)	
	Mean ± SD	CV (%)	Mean ± SD	CV (%)	Mean ± SD	CV (%)	Mean ± SD	CV (%)	Mean ± SD	CV (%)
PAI-1 +11053	-	-	-	-	-	-	-	-	-	-
TT	−3.27 ± 0.88	26.9	777.10 ± 751.91	96.8	25.49 ± 15.97	62.7	5.91 ± 2.84	48.1	12.15 ± 7.79	64.1
TG	−2.99 ± 0.98	32.8	684.05 ± 485.48	71	23.90 ± 24.69	103.3	8.92 ± 8.47	95	6.54 ± 4.56	69.7
GG	−3.07 ± 1.01	32.9	891.56 ± 1104.54	123.9	23.06 ± 19.83	86	8.14 ± 3.72	45.7	8.33 ± 3.20	38.4
p	0.464	-	0.181	-	0.945	-	0.46	-	0.053	-
TT vs. TG + GG	−3.02 ± 0.99	32.8	748.70 ± 739.61	98.8	23.60 ± 22.92	97.1	8.66 ± 7.12	82.2	7.14 ± 4.18	58.5
p	0.234	-	0.785	-	0.759	-	0.225	-	0.022	-

Note: BMD, bone mineral density; Vit. B12, plasma vitamin B12; 25-Vit. D, 25-OH (hydroxyl) vitamin D; DPD, Deoxypyridinoline; SD, standard deviation; CV, between-person coefficient of variation; * Analysis was performed by Kruskal–Wallis test in the differences of biochemical parameters between *PAI-1* gene polymorphisms in OVCF women.

Table S9. The polymerase chain reaction (PCR) restriction fragment length polymorphism (RFLP) assay.

SNPs	Polymorphism	Region	rs #	Forward Primer (5′-3′)	Reverse Primer (5′-3′)	Restriction Enzyme *
PAI-1 −844	G > A	5′-UTR	rs2227631	5′-CAG GCT CCC ACT GAT TCT AC-3′	5′-GAG GGC TCT CTT GTG TCA AC-3′	*Xho* I
PAI-1 −675	4G > 5G	5′-UTR	rs1799889	5′-CCA ACA GAG GAC TCT TGG TC-3′	5′-CAC AGA GAG AGT CTG GCC ACG-3′	*Bsl* I
PAI-1 +43	G > A	Exon 2	rs6092	5′-TGT CTT CCA GAA CGA TTC CTT CAC C-3′	5′-GTT GTC AGC TGG AGC ATG GCC-3′	*PshA* I
PAI-1 +9785	G > A	Intron 7	rs2227694	5′-TTA GCC AGG CAT GGT GGC ATG-3′	5′-CGG GCT GAG ACT ATG ACA GCT GC-3′	*Aci* I
PAI-1 +11053	T > G	3′-UTR	rs7242	5′-GCT GGG CAC GCA TCT GGC-3′	5′-TCT CCC TCC CCA GAA ACA GTG TGC-3′	*Hae* III

* All of the restriction enzymes were available from New England Biolabs (MA, USA) and we used the reaction conditions recommended by the instructions.
